# Study of the antivirus patch testing problem through optimal control modeling

**DOI:** 10.1371/journal.pone.0319916

**Published:** 2025-05-06

**Authors:** Guofang Liu, Chunlong Fu, Xiaofan Yang, Luxing Yang, Yanhua Feng, Yang Qin

**Affiliations:** 1 Department of Computer Science, Sichuan University Jinjiang College, MeiShan, Sichuan 620860, China; 2 School of Big Data and Software Engineering, Chongqing University, Chongqing 400044, China; 3 School of Information Technology, Deakin University, Melbourne, Victoria 3125, Australia; Parahyangan Catholic University, INDONESIA

## Abstract

The lag of antivirus (AV) software development relative to malware development makes it necessary to constantly release AV patches. In practice, an AV patch can be deployed on an organization’s intranet only when it passes compatibility test. In this context, a subset of hosts may be assigned to perform the test. The function of the fraction of the assigned hosts with respect to time is referred to as an AV patch testing (AVPT) policy, and the problem of finding a satisfactory AVPT policy in terms of the cost benefit is referred to as the AVPT problem. This paper addresses the AVPT problem through optimal control modeling. A new mathematical model of characterizing the evolution of the intranet’s expected state is introduced by incorporating the effect of AV patch testing. On this basis, the AVPT problem is modeled as an optimal control problem (the AVPT model). By applying the Pontryagin Maximum Principle to this model, an iterative algorithm of solving the model is presented. The usability of the algorithm, including its convergence and effectiveness, is validated. Finally, the effect of a pair of controllable factors is inspected. This work initiates the study of patch testing-related issues through optimal control modeling.

## Introduction

Malicious software, or malware, serves as the umbrella term of all kinds of computer programs that perform vicious operations [1]. Computer viruses, worms, Trojan horses, rootkits, and ransomware are typical examples of malware. Today, the development of malware has come to be a highly profitable business, with the intent of extorting money from computer users or stealing their credentials for stealing their money or digital assets. With the rapidly increasing number and variety of malware, the total amount of financial losses suffered by the whole world is increasing every year. To exemplify, it was reported that, owing to the rampage of the infamous ransomware, only in 2020 the overall ransom paid by the victims was over 412 million US dollars [2].

To mitigate the serious consequence of malware, a multitude of malware detection techniques have been developed [3, 4]. There are two different kinds of malware detection techniques: signature-based and behavioral-based. A signature-based malware detection technique can accurately identify malware with known signatures but fails when facing new malware with no known signatures. A behavioral-based malware detection technique can identify some new malware but with higher false positive rate. Antivirus (AV) software refers to software designed to detect and remove malware from computers [5]. The development of AV software depends heavily on the advance of malware detection techniques. Nowadays, there are lots of commercially available AV products.

### Motivation

With the rapid progress of software automation techniques, the development of malware is speeding up at an alarming rate. As a result, lots of new malware is emerging on a daily basis. In contrast, the development of AV software markedly lags behind that of malware. Therefore, new AV patches must be developed constantly to come up with the emerging malware. As a special kind of software patch, AV patch must be managed following the standard procedure of patch management [6].

As a critical component of AV patch management, AV patch testing aims to decide on if an AV patch is compatible with the software and applications existing in a computer [7]. Although each and every AV patch undergoes rigorous compatibility test before it is released, it is impossible for an AV patch vendor to examine the compatibility of an AV patch by exhausting all possibilities. Therefore, before an AV patch can be installed on a computer, it is typically the computer user’s obligation to test the patch compatibility comprehensively.

Consider the typical application scenario where a large-scale organization owns an intranet and has subscribed VIP services from a well-known AV vendor. This implies that at any time the organization can acquire up-to-date AV patches from the vendor. In this situation, the organization’s network administrator is responsible for testing the compatibility of the newly acquired AV patches with the software and applications deployed on the intranet. To manage the AV patch update process efficiently, the organization needs to allocate a certain amount of resources for patch testing in advance [8].

Suppose at each time point in a period of time, a subset of hosts in the intranet are assigned to participate in AV patch testing. It is easily understood that assigning a host to perform AV patch test helps reduce the likelihood of exposure of patch incompatibility, at the cost of a certain degree of performance degradation. We refer to the function of the fraction of the assigned hosts with respect to time as an *AV patch testing (AVPT) policy*. In practice, the organization needs to solve the following problem:

*AVPT problem:* Find a cost-effective AVPT policy from massive candidate AVPT policies.

To our knowledge, this problem has not been touched in literature. This paper aims to solve the AVPT problem.

### Contributions

The main contributions of this paper are sketched below.

- *Formulation and modeling of the AVPT problem*. The AVPT problem is formulated explicitly. By incorporating the effect of AV patch testing, a new mathematical model of characterizing the evolution of the intranet’s expected state is introduced. On this basis, the AVPT problem is boiled down to an optimal control problem (the AVPT model).
- *Solution of the AVPT model*. By applying the Pontryagin Maximum Principle to the AVPT model, an iterative algorithm for solving the AVPT model is presented. The usability of the algorithm, including its convergence and effectiveness, is validated through numerical experiments.
- *Further discussions*. A pair of factors that are under the control of the organization are identified. The effect of these two factors on the AVPT policy generated by the above algorithm is examined.

The remaining part of this paper is organized in this way: Section II reviews the related work. Section III establishes the AVPT model. Section IV gives an algorithm of solving the AVPT model and validates its usability. Section V inspects the effect of a pair of controllable factors. Finally, this work is summarized by section VI.

## Related work

This section is dedicated to reviewing the related work.

### Modeling of malware propagation

Infectious diseases can spread in populations. To evaluate the prevalence of an infectious disease as well as the effect of a treatment measure, in 1927 Kermack and McKendrick introduced a differential system, termed the susceptible-infected-recovered (SIR) model, to capture the spreading process of an epidemic with no recurrence [9]. Later, in 1933 they suggested a differential system, termed the susceptible-infected-susceptible (SIS) model, to characterize the spreading process of an endemic or a recurrent epidemic [10]. Their seminal work laid foundation for the birth of epidemic dynamics [11].

In 1964 Goffman and Newill suggested to view any propagation phenomenon as an ‘epidemic’ and investigate its propagation laws through epidemic modeling [12]. In response to this suggestion, in 1991-1993 Kephart and White advised the earliest malware propagation models to evaluate the impact of malware propagation as well as the effect of AV patch release [13, 14]. Their work laid foundation for the birth of malware propagation dynamics. Since then, many malware propagation models, ranging from ordinary models [15, 16] to delayed models [17, 18], and from models on homogeneous networks [15, 16] and models on inhomogeneous networks [19, 20] to models on arbitrary networks [21, 22], have been proposed.

In practice, there are two different kinds of AV patch distribution mechanism: *centralized* and *decentralized*. With regard to the former distribution mechanism, all customers of an AV vendor acquire AV patches by downloading them directly from the vendor’s website. See [13–22] for some malware propagation models under the centralized AV patch distribution mechanism. The major defect of this distribution mechanism lies in the limited bandwidth available for AV patch downloading. When it comes to the latter distribution mechanism, only a small fraction of customers acquire AV patches through direct patch downloading, while all the remaining customers acquire them through automated AV patch forwarding over the Internet [23]. In this situation, malware and AV patches can propagate simultaneously over the same or different networks. See [24–28] for some malware-patch mixed propagation models under the decentralized AV patch distribution mechanism. This distribution mechanism overcomes the limitation on the bandwidth available for downloading AV patches.

In all the above-mentioned malware propagation models, the AV patch downloading rate is assumed to be time-invarying. Additionally, in all the above-mentioned malware-patch mixed propagation models, the AV patch forwarding rate is assumed to be time-invarying. In practice, these rates can be adjusted flexibly.

### Optimal control of malware propagation

In the presence of a sole decision-maker, optimal control theory aims to find a policy governing a dynamical system so that the decision-maker’s payoff is optimized [29]. In the presence of multiple decision-makers (players), differential game theory aims to find a policy portfolio governing a dynamical system so that each and every player is satisfied with his own payoff [30]. By introducing one or more control variables into a malware propagation model, the control problem of malware propagation can be modeled as an optimal control problem or a differential game problem.

Based on a malware propagation model on a mobile wireless network, [31] considered the tradeoff between the network’s security risk and the bandwidth consumption of patching. Through optimal control modeling and analysis, the authors acquired a better tradeoff policy. Based on a malware propagation model on an inhomogeneous mobile network, [32] attempted to minimize the overall cost yielded by malware propagation and patching. Again through optimal control modeling, the authors acquired a better centralized AV distribution policy. In the presence of a strategic, intelligent malware maker and based on a malware-patch mixed propagation model, [33] addressed the downloading of AV patches. Through differential game modeling, the authors acquired a better AV patch downloading policy. Recently, based on a delayed malware propagation model on a mobile wireless sensor network, [34] addressed the minimization of the sum of the amount of losses caused by malware propagation and the cost used for patching. Through optimal control modeling of delayed dynamic system, the authors obtained a better centralized AV patch distribution strategy.

In practice, a new AV patch must be tested before it is installed. However, none of the above work considered the effect of AV patch testing.

### Software patch management

[35] offered an excellent literature review on software patch management. In particular, the authors pointed out that one of the biggest challenges faced by patch testing is the lack of a proper automated test strategy. This may be attributed to multiple aspects, ranging from complex patch dependencies [36, 37] to expenive cost for establishing test environment [38, 39]. As a result, most of the current patch testing are conducted manually to avoid unexpected system breakdown. However, the poor test quality in manual patch testing increases the risk of system performance degradation. In recent years some automated patch testing techniques have been developed [40–42]. We believe that, in the near future, the accuracy of automated patch testing will be enhanced greatly. This is the foundation on which the present work is based.

In a patch management process involving a software vendor and a firm, the vendor wishes to determine the best patch-release policy, and the firm wants to select the best patch-update policy. In the context of time-driven patch release and update, [8] developed a game model to characterize the strategic interaction between the vendor and the firm. Under the centralized path-release and update mechanism, the authors showed that the social loss is minimized when the patch-release and update cycles are synchronized. Under the decentralized path-release and update mechanism, the authors gave a sufficient condition for a pair of patch-release and update policies to achieve equilibrium. This work is somewhat related to ours. However, this work has no relationship with patch testing.

### Novelties of the present paper

Albert Einstein once said: “To raise new questions, new possibilities, to regard old problems from a new angle, requires creative imagination and marks real advance in science.” The first novelty of the present paper is to propose a new, valuable problem (i.e., the AVPT problem). First, this problem has never been touched in literature. Second, the problem is worth studying from the perspective of patch management, because its solution could offer insight into AV patch testing.

The second novelty of the present paper is to introduce a new malware propagation model. To our knowledge, none of existing malware propagation models took the effect of AV patch testing into account. In our proposed malware propagation model, the performance degradation caused by AV patch testing and that caused by the exposed patch incompatibility are well characterized. This work lays foundation for the theoretical study of AV patch testing.

The third novelty of the present paper is to study the proposed AVPT problem through mathematical modeling. To our knowledge, all the existing researches on patch testing lack an in-depth mathematical treatment. Based on our proposed malware propagation model, in this paper the AVPT problem is successfully reduced to an optimal control problem. As a result, the AVPT problem is resolved successfully by applying optimal control theory to the proposed optimal control problem.

## Modeling of the AVPT problem

This section is devoted to the modeling of the AVPT problem proposed in the first section.

### Formalization of AVPT policy

Consider the AVPT problem. Suppose the organization possesses an intranet of *N* hosts and has subscribed VIP services from an AV vendor. This implies that, whenever necessary, the organization can acquire up-to-date AV patches from the AV vendor. To safeguard the intranet against malware attack, the organization’s network administrator needs to timely test and deploy newly acquired AV patches. In this context, it is necessary to evaluate the effect of AV patch testing in advance. Let [0,T] denote the future time horizon preset for the evaluation. We refer to *T* as the *test evaluation period*.

For our purpose, we need to formalize an AVPT policy. Let *x*(*t*) denote the fraction of hosts assigned to perform AV patch test at time *t*. Then, 0≤x(t)≤1, 0≤t≤T. We refer to the function *x* defined on the interval [0,T] as an *AV patch testing (AVPT) policy*.

Assigning a host to perform AV patch test would lead to a certain degree of performance degradation of the organization. This is because the host cannot be assigned to perform any other operations at the same time. Define the *basic test cost*, denoted $\omega_{1}$, as the per-unit-time cost yielded by the performance degradation caused by assigning a host to perform AV patch test. Then, an AVPT policy *x* is implemented at the test cost

$$C_{1}(x)=\omega_{1}\;N\int_{0}^{T}x(t)dt.$$ (1)

Now, consider the feasibility of an AVPT policy. On the one hand, to guarantee the organization’s normal operation, the fraction of the assigned hosts must be small. Suppose all *x*(*t*) are bounded by $\overset{―}{x}$. We refer to $\overset{―}{x}$ as the *maximal host fraction*. On the other hand, to guarantee smooth transition of the assigned hosts, the change in the fraction of the assigned hosts must be small. Hence, in what follows assume an AVPT policy is feasible only if it is piecewise continuous. Let PC[0,T] denote the set of piecewise continuous functions defined on [0,T]. Combining the above discussions, we get that an AVPT strategy is feasible if and only if it falls into the set

$${𝒳}_{T,\overset{―}{x}}=\{x\in PC[0,T]:0\leq x(t)\leq \overset{―}{x},0\leq t\leq T\}.$$ (2)

### Expected state of the intranet and its evolution

For our purpose, it is essential to define the intranet’s expected state and model its evolution.

At any time point in the future time interval [0,T], each and every host in the intranet is either *operable*, i.e., working normally, or *inoperable*, i.e., not working normally. In what follows we make the following ideal assumptions:

(*A*_1_) If a host is operable at a time point, this can be attributed to one of two possibilities: (i) The installed AV patches are compatible perfectly with the system and applications deployed on the host. (ii) Although the installed AV patches are not perfectly compatible, this imperfection doesn’t hinder the host’s normal operation at this time point.
(*A*_2_) If a host doesn’t operable at a time point, this can be exclusively attributed to the possibility that (i) the installed AV patches are not perfectly compatible, and (ii) this imperfection impedes the host’s normal operation at this time point.

Furthermore, at any time point each and every operable host may be *susceptible*, i.e., not currently infected with malware, or *compromised*, i.e., currently infected with malware. Combining the above discussions, at any time point each and every host in the intranet is in one of three possible states: susceptible, compromised, and inoperable.

Let *X*_1_(*t*) (resp. *X*_2_(*t*), *X*_3_(*t*)) denote the number of susceptible (resp. compromised, inoperable) hosts at time *t*. As $X_{1}(t)+X_{2}(t)+X_{3}(t)\equiv N$, we define the *state* of the intranet at time *t* as the vector

$$X(t)=(X_{1}(t),X_{2}(t)).$$ (3)

In practice, *X*(*t*) is a random vector, which cannot be predicted accurately. Instead, below let us define the expected state of the intranet.

Let *S*(*t*) (resp. *C*(*t*), *I*(*t*)) denote the expected number of susceptible (resp. compromised, inoperable) hosts at time *t*. As S(t)+C(t)+I(t)≡N, we define the *expected state* of the intranet at time *t* as the vector

E(t)=(S(t),C(t)). (4)

In practice, *E*(*t*) can be predicted accurately.

To capture the evolution of the expected state of the intranet over time, below let us introduce several reasonable assumptions. Here. the term *rate* refers to probability per unit time.

(*A*_3_) Owing to the impact of malware attack coming from outside of the organization, each and every susceptible host gets infected at any time at the constant rate α (the *external infection rate*).
(*A*_4_) Owing to the impact of malware attack coming from a compromised host, each and every susceptible host gets infected at any time at the constant rate β (the *internal infection rate*).
(*A*_5_) Owing to the influence of the installed AV patches, each and every compromised host becomes susceptible at any time at the constant rate γ (the *disinfection rate*).
(*A*_6_) Owing to the impact of exposure of incompatibility of the installed AV patches, each and every operable host becomes inoperable at any time at the rate δ(z), where *z* stands for the fraction of hosts assigned to perform AV patch test at that time. The function δ is referred to as the *incompatibility rate function*. By the definition, δ is decreasing.
(*A*_7_) Owing to the influence of reinstalling systems on inoperable hosts, each and every inoperable host becomes susceptible at any time at the constant rate θ (the *repair rate*).
(*A*_8_) At the initial time, the intranet has just been fully disinfected and restarted successfully. This implies S(0)=N,C(0)=0.

Under the above assumptions, the evolution of the expected state of the intranet obeys the following differential system:

$$\{\frac{dS(t)}{dt}=-[\alpha +\beta C(t)+\delta (x(t))]S(t)+\gamma C(t)\;\;+\theta [N-S(t)-C(t)],0\leq t\leq T,\;\frac{dC(t)}{dt}=[\alpha +\beta C(t)]S(t)-[\gamma +\delta (x(t))]C(t),\;0\leq t\leq T,\;S(0)=N,C(0)=0.$$ (5)

**Fig 1:**
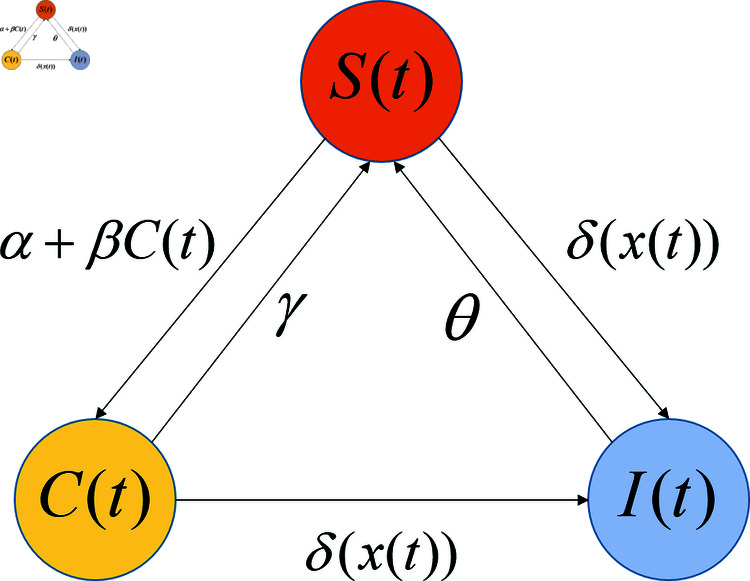
Diagram of the system (5).

Fig 1 gives a diagram of this system.

### Optimal control modeling of the AVPT problem

We are ready to finish the modeling of the AVPT problem.

The exposure of incompatibility of the AV patches installed on an operable host would render the host inoperable and lead to a certain degree of performance degradation of the organization. Define the *basic incompatibility cost*, denoted $\omega_{2}$, as the per-unit-time cost yielded by the performance degradation caused by the exposed incompatibility of the AV patches installed on an host. Then, an AVPT policy *x* is implemented at the incompatibility cost

$$C_{2}(x)=\omega_{2}\int_{0}^{T}[N-S(t)-C(t)]dt.$$ (6)

A susceptible host can bring a certain amount of benefit. This is because the host is not only working normally but not infected with malware. Define the *basic security benefit*, denote $\omega_{3}$, as the per-unit-time benefit yielded by a susceptible host. Then, the benefit brought by an AVPT policy *x* is estimated to be

$$B(x)=\omega_{3}\int_{0}^{T}S(t)dt.$$ (7)

In view of Eq. 1, the cost benefit of implementing an AVPT policy *x* is estimated to be

$$J(x)=B(x)-C_{1}(x)-C_{2}(x)=\omega_{3}\int_{0}^{T}S(t)dt\;\;-\omega_{1}N\int_{0}^{T}x(t)dt-\omega_{2}\int_{0}^{T}[N-S(t)-C(t)]dt.$$ (8)

Combining the previous discussions, in what follows we reduce the AVPT problem to the following optimal control problem:

$$\max_{x\in {𝒳}_{T,\overset{―}{x}}}J(x)=\omega_{3}\int_{0}^{T}S(t)dt-\omega_{1}N\int_{0}^{T}x(t)dt\;\;\;\;\;\;\;\;-\omega_{2}\int_{0}^{T}[N-S(t)-C(t)]dt\;s.t.\{\frac{dS(t)}{dt}=-[\alpha +\beta C(t)+\delta (x(t))]S(t)+\gamma C(t)\;\;\;\;+\theta [N-S(t)-C(t)],0\leq t\leq T,\;\frac{dC(t)}{dt}=[\alpha +\beta C(t)]S(t)-[\gamma +\delta (x(t))]C(t),\;\;\;\;0\leq t\leq T,\;S(0)=N,C(0)=0.$$ (9)

We refer to the problem (9) as the *AVPT model*. Each instance of the AVPT model (AVPT instance, for short) is captured by a 11-tuple of the form

$$𝕄=(N,T,\overset{―}{x},\alpha ,\beta ,\gamma ,\theta ,\delta ,\omega_{1},\omega_{2},\omega_{3}).$$ (10)

## Solution of the AVPT model

This section is dedicated to solving the AVPT model established in the preceding section. First, solving the AVPT model is partly reduced to solving a system. Second, an iterative algorithm for solving the system is given. For detailed knowledge on optimal control theory, see [29].

### Optimality system

The Hamiltonian function for the AVPT model reads

$$H(S,C,x,\lambda ,\mu )\;=\omega_{3}S-\omega_{1}Nx-\omega_{2}(N-S-C)\;+\lambda \{-[\alpha +\beta C+\delta (x)]S+\gamma C+\theta (N-S-C)\}\;+\mu \{(\alpha +\beta C)S-[\gamma +\delta (x)]C\}.$$ (11)

Here, (λ,μ) stands for the adjoint of *H*.

Suppose *x* is an optimal AVPT policy for the AVPT model (9). Let (S,C) be the solution to the associated expected state evolutionary model (5). It follows from the Pontryagin Maximum Principle [29] that there exists an adjoint funtion (λ,μ) such that

$$\{\frac{d\lambda (t)}{dt}=-\frac{\partial H(S(t),C(t),x(t),\lambda (t),\mu (t))}{\partial S},0\leq t\leq T,\;\frac{d\mu (t)}{dt}=-\frac{\partial H(S(t),C(t),x(t),\lambda (t),\mu (t))}{\partial C},0\leq t\leq T,\;\lambda (T)=\mu (T)=0.$$ (12)

Through direct calculations, we get that

$$\{\frac{d\lambda (t)}{dt}=[\alpha +\theta +\beta C(t)+\delta (x(t))]\lambda (t)-[\alpha +\beta C(t)]\mu (t)\;\;-\omega_{3}-\omega_{2},0\leq t\leq T,\;\frac{d\mu (t)}{dt}=[\beta S(t)+\theta -\gamma ]\lambda (t)+[\delta (x(t))+\gamma -\beta S(t)]\mu (t)\;\;-\omega_{2},0\leq t\leq T,\;\lambda (T)=\mu (T)=0.$$ (13)

Again by the Pontryagin Maximum Principle [29], we have

$$x(t)\in \mathrm{\backslash arg}\max_{0\leq \tilde{x}\leq \overset{―}{x}}H(S(t),C(t),\tilde{x},\lambda (t),\mu (t)),0\leq t\leq T.$$ (14)

Let

$$h_{t}(z)=[\lambda (t)S(t)+\mu (t)C(t)]\delta (z)+\omega_{1}Nz.$$ (15)

It follows from Eqs. 14 and through simple algebraic calculations that

$$x(t)\in \mathrm{\backslash arg}\min_{0\leq \tilde{x}\leq \overset{―}{x}}h_{t}(\tilde{x}),0\leq t\leq T.$$ (16)

Combining Eqs. 5, 13, and 16, we get the optimality system for the AVPT model (9), which is given below.

$$\{\frac{dS(t)}{dt}=-[\alpha +\beta C(t)+\delta (x(t))]S(t)+\gamma C(t)\;\;+\theta [N-S(t)-C(t)],0\leq t\leq T,\;\frac{dC(t)}{dt}=[\alpha +\beta C(t)]S(t)-[\gamma +\delta (x(t))]C(t),0\leq t\leq T,\;\frac{d\lambda (t)}{dt}=[\alpha +\theta +\beta C(t)+\delta (x(t))]\lambda (t)-[\alpha +\beta C(t)]\mu (t)\;\;-\omega_{3}-\omega_{2},0\leq t\leq T,\;\frac{d\mu (t)}{dt}=[\beta S(t)+\theta -\gamma ]\lambda (t)+[\delta (x(t))+\gamma -\beta S(t)]\mu (t)\;\;-\omega_{2},0\leq t\leq T,\;x(t)\in \mathrm{\backslash arg}\min_{0\leq \tilde{x}\leq \overset{―}{x}}h_{t}(\tilde{x}),0\leq t\leq T,\;S(0)=N,C(0)=0,\lambda (T)=\mu (T)=0.$$ (17)

In what follows we view the optimality system (17) as a system in *x*. Here, *S*, *C*, λ, and μ are viewed as auxiliary functions.

An optimal control for the AVPT model (9) must be a solution to the system (17). But the converse is untrue.

### Solving the optimality system

See Ref. [43] for a standard method of solving open-loop, deterministic optimal control problems. On this basis, an algorithm of solving the AVPT model (9), termed the AVPT algorithm, is formulated in Algorithm 1. When performing the algorithm, a sequence of feasible AVPT policies are generated. The algorithm terminates if and only if the generated sequence converges in terms of the preset convergence error. If this is the case, the algorithm returns the finally generated AVPT policy.

**Algorithm 1** AVPT

**Input:** an AVPT instance $𝕄=(N,T,\overset{―}{x},\alpha ,\beta ,\gamma ,\theta ,\delta ,\omega_{1},\omega_{2},$ $\omega_{3})$, a convergence error ϵ.

**Output:** an AVPT policy *x*.

1: k←0; $x^{\left(0\right)}\leftarrow 0$;

2: **repeat**

3:   k←k+1;

4:   forwardly caculate (S,C) using Eqs. 5 with $x\leftarrow x^{\left(k-1\right)}$;

$\left(S^{\left(k\right)},C^{\left(k\right)})\leftarrow (S,C\right)$;

5:   backwardly caculate (λ,μ) using Eqs. 13 with $x\leftarrow x^{\left(k-1\right)}$

and $\left(S,C)\leftarrow (S^{\left(k\right)},C^{\left(k\right)}\right)$; $\left(\lambda^{\left(k\right)},\mu^{\left(k\right)})\leftarrow (\lambda ,\mu \right)$;

6:   caculate *x* by solving the minimization problems (16) with

$\left(S,C)\leftarrow (S^{\left(k\right)},C^{\left(k\right)}\right)$ and $\left(\lambda ,\mu )\leftarrow (\lambda^{\left(k\right)},\mu^{\left(k\right)}\right)$; $x^{\left(k\right)}\leftarrow x$;

7: **until** $\underset{0\leq t\leq T}{\sup}|x^{\left(k\right)}(t)-x^{\left(k-1\right)}(t)|<\epsilon$;

8: **return** *x*^(*k*)^.

Before the AVPT algorithm can be used to solve the AVPT model, a pair of questions must be answered beforehand. The first question is: Does the AVPT algorithm converge? (equivalently, does the sequence of AVPT policies generated by the AVPT algorithm converge?) After all, the AVPT algorithm returns a feasible AVPT policy if and only if it converges. The second question is: In the case where the AVPT algorithm converges, is the AVPT algorithm effective? (equivalently, is the AVPT policy generated by the AVPT algorithm cost-effective)? Now, let us answer these two questions through extensive numerical experiments.

**Experiment 1.** *Consider the AVPT instance ${𝕄}_{1}=(N,T,\overset{―}{x},\alpha ,\beta ,\gamma ,\theta ,\delta ,\omega_{1},\omega_{2},\omega_{3})$, where *N* = 1000, *T* = 20, $\overset{―}{x}=0.1$, α=0.05, β=0.001, γ=0.8, θ=0.1, $\delta (z)=e^{-100z}$, $\omega_{1}=10$, $\omega_{2}=30$, $\omega_{3}=80$.*

**Fig 2:**
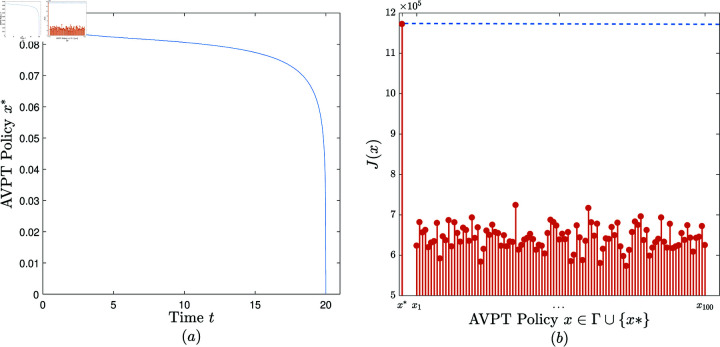
The experimental results obtained in Experiment 1.

1. Convergence. When performing the AVPT algorithm on $\left({𝕄}_{1},10^{-6}\right)$, it is observed that the algorithm terminates in six iterations, returning an AVPT policy, denoted x^*^ and plotted in Fig. 2(a).
2. Effectiveness. Randomly and uniformly generate a set of 100 feasible AVPT policies, denoted $\Gamma =\{x_{1},\ldots ,x_{100}\}$. Fig. 2(b) exhibits *J*(*x*) versus *x*, $x\in \{x^{*}\}\cup \Gamma$. It is observed that *J*(x^*^)>*J*(*x*) for all x∈Γ. That is, x^*^ is superior to all the 100 AVPT policies in Γ in terms of the cost benefit.

**Experiment 2.** *Consider the AVPT instance ${𝕄}_{2}=(N,T,\overset{―}{x},\alpha ,\beta ,\gamma ,\theta ,\delta ,\omega_{1},\omega_{2},\omega_{3})$, where *N* = 2000, *T* = 30, $\overset{―}{x}=0.2$, α=0.05, β=0.0005, γ=0.8, θ=0.1, $\delta (z)=\frac{1}{500z+1}$, $\omega_{1}=20$, $\omega_{2}=50$, $\omega_{3}=100$.*

**Fig 3:**
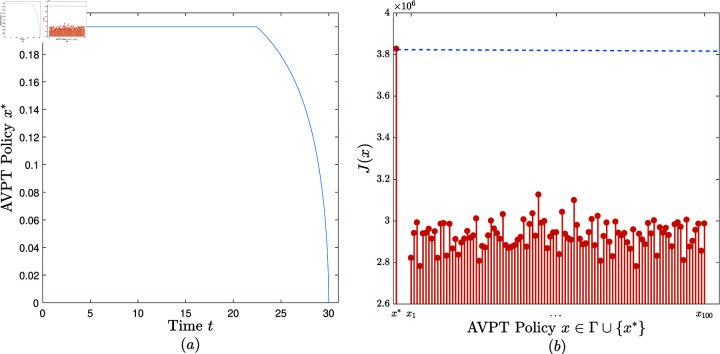
The experimental results obtained in Experiment 2.

1. Convergence. When performing the AVPT algorithm on $\left({𝕄}_{2},10^{-6}\right)$, it is observed that the algorithm terminates in four iterations, returning an AVPT policy, denoted x^*^ and plotted in Fig. 3(a).
2. Effectiveness. Randomly and uniformly generate a set of 100 feasible AVPT policies, denoted $\Gamma =\{x_{1},\ldots ,x_{100}\}$. Fig. 3(b) exhibits J(x) versus x, $x\in \{x^{*}\}\cup \Gamma$. It is observed that *J*(x^*^)>*J*(*x*), x∈Γ. That is, x* outperforms all the 100 AVPT policies in Γ in terms of the cost benefit.

**Experiment 3.** *Consider the AVPT instance ${𝕄}_{3}=(N,T,\overset{―}{x},\alpha ,\beta ,\gamma ,\theta ,\delta ,\omega_{1},\omega_{2},\omega_{3})$, where *N* = 3000, *T* = 50, $\overset{―}{x}=0.3$, α=0.05, β=0.0001, γ=0.8, θ=0.1, $\delta (z)=(1-3z{)}^{7}$, $\omega_{1}=30$, $\omega_{2}\;=\;70$, $\omega_{3}=180$.*

**Fig 4:**
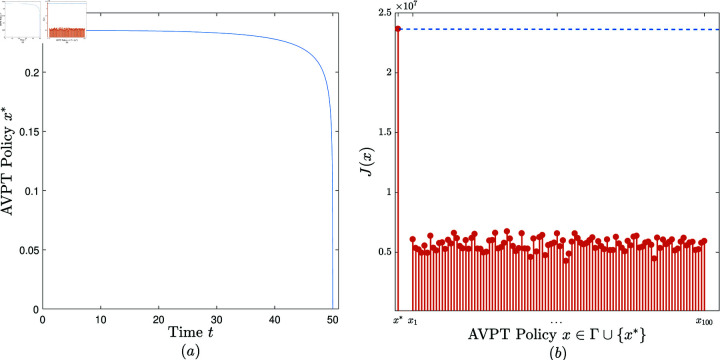
The experimental results obtained in Experiment 3.

Convergence. When performing the AVPT algorithm on $\left({𝕄}_{3},10^{-6}\right)$, it is observed that the algorithm terminates in three iterations, returning an AVPT policy, denoted x^*^ and plotted in Fig 4a.
2. Effectiveness. Randomly and uniformly generate a set of 100 feasible AVPT policies, denoted $\Gamma =\{x_{1},\ldots ,x_{100}\}$. Fig 4b exhibits J(x) versus x, $x\in \{x^{*}\}\cup \Gamma$. It is observed that *J*(x^*^)>*J*(*x*), x∈Γ. That is, x* beats all the 100 AVPT policies in Γ in terms of the cost benefit.

Additionally, 10000 similar experiments have been conducted. In each of these experiments, similar phenomena are observed. Hence, it is concluded that the AVPT algorithm converges almost surely and that the resulting AVPT policy outperforms most other feasible AVPT policies in terms of the cost benefit almost surely. That is, the two previously proposed questions are answered positively. Therefore, it is concluded that the AVPT algorithm can be used to solve the AVPT model successfully. Consequently, the AVPT policy generated by the AVPT algorithm is strongly recommended.

Look at Ref. [44]. A dynamic programming method of solving optimal control problems was presented. It is concluded that, generally speaking, our method is significantly superior to this method in terms of the cost benefit, the time cost, and the smoothness of the control strategy.

## Further discussions

This section is devoted to inspecting the effect of some key factors on the AVPT policy obtained by the AVPT algorithm.

### Classification of the relevant factors

First, the eleven factors involved in the AVPT instance (10) can be categorized as three classes, which are listed below.

- *Class 1:* Known factors. There is only one such factor: the intranet size *N*.
- *Class 2:* Controllable factors. There are two factors that are under the control of the organization: the test evaluation period *T* and the maximal host fraction $\overset{―}{x}$. In practice, *T* is preset by the organization based on the evaluation requirement, $\overset{―}{x}$ is determined by the organization based on the demanded performance level.
- *Class 3:* Uncontrollable but estimable factors. There are eight such factors: the external infection rate α, the internal infection rate β, the disinfection rate γ, the incompatibility rate function δ, the repair rate θ, the basic test cost $\omega_{1}$, the basic incompatibility cost $\omega_{2}$, and the basic security benefit $\omega_{3}$.

In practice, α and β can be estimated by averaging the historical data of the relevant malware infection rate, γ can be estimated by averaging the historical data of the AV patch installation rate, θ can be estimated by averaging the historical data of system installation rate, δ can be approximated by fitting the historical data about the probability distribution of the exposure of AV patch incompatibility, $\omega_{1}$ and $\omega_{2}$ can be estimated based on the degree of performance degradation caused by a computer in downtime, and $\omega_{3}$ can be estimated based on the economic losses caused by a compromised computer.

In practice, when all the above-mentioned factors have been determined or estimated, an AVPT instance takes shape. By performing the AVPT algorithm on this instance (as well as a given convergence error), a cost-effective AVPT policy is generated, which is recommended to the associated organization.

### Effect of a pair of controllable factors

In the previous subsection, we stated that there are a pair of factors, i.e., the test evaluation period and the maximal host fraction, that are under the control of the organizations. Now, let us inspect the effect of these factors on the AVPT policy generated by the AVPT algorithm through numerical experiments. First, examine the effect of the test evaluation period.

**Experiment 4.** *Let 𝒯={10,20,…,100}.*

**Fig 5:**
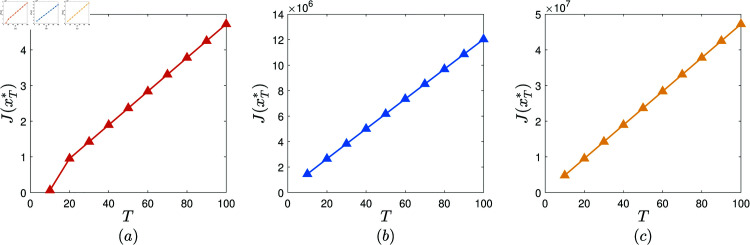
The experimental results obtained in Experiment 4.

1. Consider the collection of AVPT instances: ${𝕄}_{1}^{T}=(N,T,\overset{―}{x},\alpha ,\beta ,\gamma ,\theta ,\delta ,\omega_{1},\omega_{2},\omega_{3})$, where *N* = 1000, $\overset{―}{x}=0.1$, α=0.05, β=0.001, γ=0.8, θ=0.1, $\delta (z)=e^{-100z}$, $\omega_{1}=10$, $\omega_{2}=30$, $\omega_{3}=80$, and T∈𝒯. Let $x_{T}^{*}$ denote the AVPT policy generated by performing the AVPT algorithm on $\left({𝕄}_{1}^{T},10^{-6}\right)$. Fig. 5(a) displays $J(x_{T}^{*})$ versus *T*, T∈𝒯. It is observed that $J(x_{T}^{*})$ increases rapidly with the increase of *T*.
2. Consider the collection of AVPT instances: ${𝕄}_{2}^{T}=(N,T,\overset{―}{x},\alpha ,\beta ,\gamma ,\theta ,\delta ,\omega_{1},\omega_{2},\omega_{3})$, where *N* = 2000, $\overset{―}{x}=0.2$, α=0.05, β=0.0005, γ=0.8, θ=0.1, $\delta (z)=\frac{1}{500z+1}$, $\omega_{1}=20$, $\omega_{2}=50$, $\omega_{3}=100$, and T∈𝒯. Let $x_{T}^{*}$ denote the AVPT policy generated by performing the AVPT algorithm on $\left({𝕄}_{2}^{T},10^{-6}\right)$. Fig. 5(b) exhibits $J(x_{T}^{*})$ versus *T*, T∈𝒯. It is observed that $J(x_{T}^{*})$ increases rapidly with the increase of *T*.
3. Consider the collection of AVPT instances: ${𝕄}_{3}^{T}=(N,T,\overset{―}{x},\alpha ,\beta ,\gamma ,\theta ,\delta ,\omega_{1},\omega_{2},\omega_{3})$, where *N* = 3000, $\overset{―}{x}=0.3$, α=0.05, β=0.0001, γ=0.8, θ=0.1, $\delta =(1-3z{)}^{7}$, $\omega_{1}=30$, $\omega_{2}=70$, $\omega_{3}=180$, and T∈𝒯. Let $x_{T}^{*}$ denote the AVPT policy generated by performing the AVPT algorithm on $\left({𝕄}_{3}^{T},10^{-6}\right)$. Fig. 5(c) depicts $J(x_{T}^{*})$ versus *T*, T∈𝒯. It is observed that $J(x_{T}^{*})$ increases rapidly with the increase of *T*.

We have conducted 100 similar experiments. In each of these experiments, similar phenomenon is observed. Therefore, we conclude that, almost surely, the cost benefit of the AVPT policy generated by the AVPT algorithm increases rapidly with the increase of the test evaluation period. In practice, the organization is suggested to properly extend the test evaluation period to enhance the cost benefit of the AVPT policy generated by performing the AVPT algorithm.

Second, investigate the effect of the maximal host fraction.

**Experiment 5.** *Let 𝒳={0.1,0.11,…,0.3}.*

**Fig 6:**
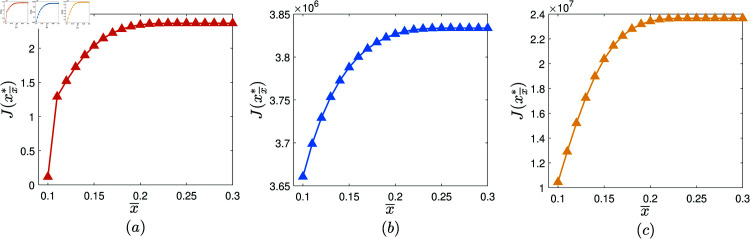
The experimental results obtained in Experiment 5.

1. Consider the collection of AVPT instances: ${𝕄}_{1}^{\overset{―}{x}}=(N,T,\overset{―}{x},\alpha ,\beta ,\gamma ,\theta ,\delta ,\omega_{1},\omega_{2},\omega_{3})$, where *N* = 1000, *T* = 20, α=0.05, β=0.001, γ=0.8, θ=0.1, $\delta (z)=e^{-100z}$, $\omega_{1}=10$, $\omega_{2}=30$, $\omega_{3}=80$, and $\overset{―}{x}\in 𝒳$. Let $x_{\overset{―}{x}}^{*}$ denote the AVPT policy generated by performing the AVPT algorithm on $\left({𝕄}_{1}^{\overset{―}{x}},10^{-6}\right)$. Fig. 6(a) shows $J(x_{\overset{―}{x}}^{*})$ versus $\overset{―}{x}$, $\overset{―}{x}\in 𝒳$. It is observed that, with the increase of $\overset{―}{x}$, $J(x_{\overset{―}{x}}^{*})$ first increases rapidly then flattens out.
2. Consider the collection of AVPT instances: ${𝕄}_{2}^{\overset{―}{x}}=(N,T,\overset{―}{x},\alpha ,\beta ,\gamma ,\theta ,\delta ,\omega_{1},\omega_{2},\omega_{3})$, where *N* = 2000, *T* = 30, α=0.05, β=0.0005, γ=0.8, θ=0.1, $\delta (z)=\frac{1}{500z+1}$, $\omega_{1}=20$, $\omega_{2}=50$, $\omega_{3}=100$, and $\overset{―}{x}\in 𝒳$. Let $x_{\overset{―}{x}}^{*}$ denote the AVPT policy generated by performing the AVPT algorithm on $\left({𝕄}_{2}^{\overset{―}{x}},10^{-6}\right)$. Fig. 6(b) displays $J(x_{\overset{―}{x}}^{*})$ versus $\overset{―}{x}$, $\overset{―}{x}\in 𝒳$. It is observed that, with the increase of $\overset{―}{x}$, $J(x_{\overset{―}{x}}^{*})$ first increases rapidly then flattens out.
3. Consider the collection of AVPT instances: ${𝕄}_{3}^{\overset{―}{x}}=(N,T,\overset{―}{x},\alpha ,\beta ,\gamma ,\theta ,\delta ,\omega_{1},\omega_{2},\omega_{3})$, where *N* = 3000, *T* = 50, α=0.05, β=0.0001, γ=0.8, θ=0.1, $\delta (z)=(1-3z{)}^{7}$, $\omega_{1}=30$, $\omega_{2}=70$, $\omega_{3}=180$, and $\overset{―}{x}\in 𝒳$. Let $x_{\overset{―}{x}}^{*}$ denote the AVPT policy generated by performing the AVPT algorithm on $\left({𝕄}_{3}^{\overset{―}{x}},10^{-6}\right)$. Fig. 6(c) displays $J(x_{\overset{―}{x}}^{*})$ versus $\overset{―}{x}$, $\overset{―}{x}\in 𝒳$. It is observed that, with the increase of $\overset{―}{x}$, $J(x_{\overset{―}{x}}^{*})$ first increases rapidly then flattens out.

We have conducted 100 similar experiments. In each of these experiments, similar phenomenon is observed. Therefore, we conclude that, almost surely, the cost benefit of the AVPT policy generated by the AVPT algorithm first increases rapidly then flattens out with the increase of the maximal host fraction. In practice, the organization is suggested to properly enhance the maximal host fraction to enhance the cost benefit of the AVPT policy generated by performing the AVPT algorithm.

## Concluding remarks

This paper has proposed an AV patch testing-related problem (i.e., the AVPT problem). By taking into account the effect of AV patch testing, a novel malware propagation model has been established. On this basis, the AVPT problem has been modeled as an optimal control problem. The model has been resolved successfully, yielding a cost-effective host assignment policy.

There are some related problems that are yet to be addressed. First, in this paper it is assumed that, although a newly installed AV patch just passed the compatibility test, it may immediately exhibit incompatibility with the deployed system and applications. In practice, there typically exists a delay from the time an AV patch is tested and installed to the time the patch exhibits incompatibility. In this situation, the proposed AVPT problem may be reduced to the optimal control problem of a delayed dynamic system [45, 46]. Second, to avoid being detected and removed by AV patches, modern malware are often designed to attack AV patches, with the intent of disabling them. In this context, there exists a non-cooperative game between the malware maker and the AV vendor, and the AVPT problem can be addressed in the framework of game theory [47]. Next, the methodology developed in this paper can be applied to some other areas, ranging from the suppression of rumor spreading [48, 49], social media advertising [50, 51], and the protection of smart grid [52, 53] to the defense against advanced cyber attacks [54, 55], provided the term *compatibility* can be given with special meaning. Finally, the state evolution model (5) may be extended to stochastic differential models [56–59] or fractional differential models [60, 61].
